# Fast Principal Component Analysis of Large-Scale Genome-Wide Data

**DOI:** 10.1371/journal.pone.0093766

**Published:** 2014-04-09

**Authors:** Gad Abraham, Michael Inouye

**Affiliations:** Medical Systems Biology, Department of Pathology and Department of Microbiology & Immunology, University of Melbourne, Parkville, Victoria, Australia; Pennsylvania State University, United States of America

## Abstract

Principal component analysis (PCA) is routinely used to analyze genome-wide single-nucleotide polymorphism (SNP) data, for detecting population structure and potential outliers. However, the size of SNP datasets has increased immensely in recent years and PCA of large datasets has become a time consuming task. We have developed flashpca, a highly efficient PCA implementation based on randomized algorithms, which delivers identical accuracy in extracting the top principal components compared with existing tools, in substantially less time. We demonstrate the utility of flashpca on both HapMap3 and on a large Immunochip dataset. For the latter, flashpca performed PCA of 15,000 individuals up to 125 times faster than existing tools, with identical results, and PCA of 150,000 individuals using flashpca completed in 4 hours. The increasing size of SNP datasets will make tools such as flashpca essential as traditional approaches will not adequately scale. This approach will also help to scale other applications that leverage PCA or eigen-decomposition to substantially larger datasets.

## Introduction

Principal component analysis (PCA) is a widely-used tool in genomics and statistical genetics, employed to infer cryptic population structure from genome-wide data such as single nucleotide polymorphisms (SNPs) [1], [2], and/or to identify outlier individuals which may need to be removed prior to further analyses, such as genome-wide association studies (GWAS). This is based on the fact that such population structure can confound SNP-phenotype associations, resulting in some SNPs spuriously being called as associated with the phenotype (false positives). The top principal components (PCs) of SNP data have been shown to map well to geographic distances between human populations [1], [3], thus capturing the coarse-grain allelic variation between these groups.

However, traditional approaches to computing the PCA, such as those employed by the popular EIGENSOFT suite [1], are computationally expensive. For example, PCA based on the singular value decomposition (SVD) scales as 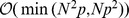 and for eigen-decomposition it is 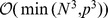 (excluding the cost of computing the covariance matrix itself which is also 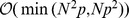), where 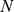 and 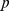 are the number of samples and SNPs, respectively. This makes it time-consuming to perform PCA on large cohorts such as those routinely being analyzed today, involving millions of assayed or imputed SNPs and tens of thousands of individuals, with this difficulty only likely to increase in the future with the availability of even larger studies.

In recent years, research into randomized matrix algorithms has yielded alternative approaches for performing PCA and producing these top PCs, while being far more computationally tractable and maintaining high accuracy relative to the traditional “exact” algorithms [4], [5]. These algorithms are especially useful when we are interested in finding only the first few principal components (PCs) of the data, as is often the case in genomic analysis.

Here we present flashpca, an efficient tool for performing PCA on large genome-wide data, based on randomized algorithms. Our approach is highly efficient, allowing the user to perform PCA on large datasets (100,000 individuals or more), extracting the top principal components while achieving identical results to traditional methods.

## Results

**Figure 1:**
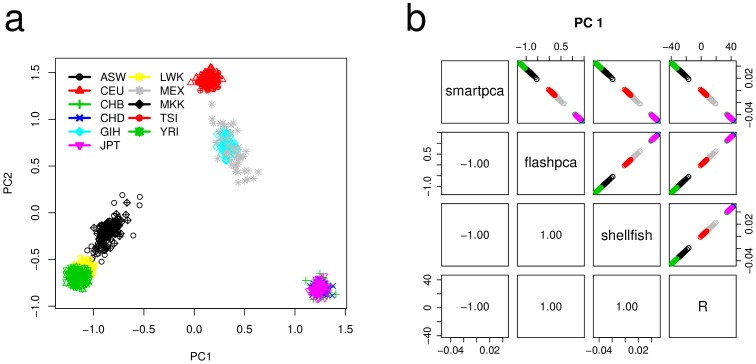
(a) The first two principal components from analyzing the HapMap3 dataset. (b) Scatter plots showing near-perfect absolute Pearson correlation (lower left-hand corner) between the 1st PCs estimated by smartpa, flashpca, shellfish, and R’s prcomp (using the standardization from Equation 4). Note that since eigenvectors are only defined up to sign, the correlations may be negative as well as positive. In addition, the scale of the PCs may differ between the methods, however, this has no bearing on the interpretation of the PCs.

First we used an LD-pruned HapMap3 genotype data [6] consisting of 957 human individuals across 11 populations assayed for 14,389 SNPs (Materials). We compared flashpca with smartpca from EIGENSOFT v4.2 (http://www.hsph.harvard.edu/alkes-price/software/) and shellfish (http://www.stats.ox.ac.uk/~davison/software/shellfish/shellfish.php). In addition, we included the R 3.0.2 [7] prcomp function which is based on SVD rather than eigen-decomposition, after replacing its original standardization with the one used by smartpca (Equation 4). The analysis of HapMap3 data revealed the expected ancestry via the first two PCs (Figure 1a), with individuals of east Asian origin (CHB, CHD, JPT) clustered in the bottom right-hand side corner, those of European origin (TSI, CEU) in the top, and those of African origin in the bottom left-hand side corner (ASW, LWK, YRI, and MKK). All methods showed close to perfect agreement on the top PC (Figure 1b), with an absolute correlation of 1.00 between the 1st PC of each pair of methods (the sign of the eigenvectors is arbitrary hence the correlation may be negative as well). The next nine PCs showed close to perfect agreement as well (see File S1 and the online documentation for results for PCs 1–10).

Next, we analyzed an LD-pruned celiac disease Immunochip dataset consisting of 16,002 individuals and 43,049 SNPs after thinning by LD [8] (Materials). We then randomly sampled subsets of the original dataset with increasing size (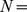
500, 1000, 2500, 5000, 7500, 10,000, 15,000), and recorded wall time for flashpca, smartpca, and shellfish performing PCA on these subsets. We also examined larger setups (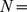
50,000, 100,000, and 150,000) by duplicating the original dataset several times as required.

Due to the substantial time required by shellfish and smartpca to complete the largest runs, we only ran flashpca on the larger datasets (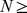
50,000) (we attempted to run shellfish on the 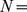
50,000 dataset but it did not complete due to running out of memory, and we stopped smartpca after 100,000 sec).

Each experiment was repeated three times. All programs used multi-threaded mode with 8 threads. All experiments were run on a machine with 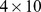-core Intel Xeon E7-4850 CPU @ 2.00 GHz with 512 GiB RAM running 64-bit Ubuntu Linux 12.04. Note that wall time here is defined as time from program start to successful exit, inclusive of any loading of data, scaling, computation of the covariance matrix, eigen-decomposition, and so on, however, the majority of the run time is taken by computing the covariance and the eigen-decomposition. smartpca was run without excluding potential population outliers.

**Figure 2:**
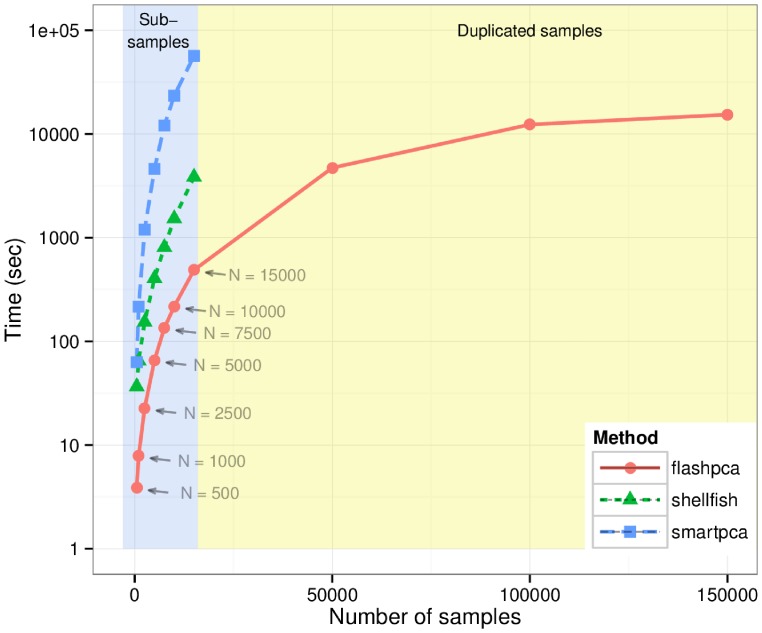
Total wall time (seconds) for flashpca versus EIGENSOFT’s smartpca and shellfish on increasing subsets of the celiac disease dataset, employing multi-threaded mode (8 threads), using 43,049 SNPs. shellfish did not complete PCA for the 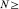
50,000 subsets, and smartpca was stopped after 100,000 sec.The results shown are averages over 3 runs. Results for 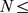
15,000 are based on subsamples of the original dataset 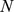 = 16,002 (light blue background), whereas results for 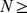
50,000 are based on duplicating the original samples (light yellow background).

Figure 2 shows that flashpca was substantially faster than either smartpca or shellfish: for analysis of 15,000 samples, flashpca took an average of 8 minutes, whereas smartpca required an average of almost 17 h (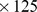 slower). Examining the large datasets, flashpca was able to analyze a dataset of 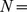
150,000 in ∼4 h, which would not be sufficient time for smartpca to complete a PCA on 10,000 samples, as 6.5 h were required for that. While shellfish was also substantially faster than smartpca, it was still considerably slower than flashpca when the number of individuals was large, with a run time of ∼1h for 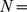
15,000 (and did not complete for subsets with 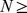
50,000).

Importantly, the time taken by flashpca to compute PCA on 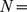
150,000 did not differ much from the time taken on the 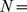
100,000 subset; this is because flashpca automatically transposes the data when 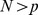, as the PCA can be computed on the original data or its transpose with only minor modifications to the algorithm; computing the 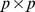 covariance matrix and its eigen-decomposition has lower computational complexity than using the 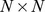 covariance matrix, for the same values of 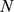 and 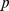, hence when 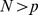 the main computational cost will not grow substantially with 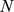.

## Discussion

Principal component analysis is an important tool in genomics for discovery of population structure or other latent structure in the data, such as batch effects. Early approaches such as smartpca from EIGENSOFT have proven useful for this goal and have been widely used for analysis of SNP datasets. However, many current datasets assay tens of thousands of individuals, making traditional approaches extremely time consuming. In contrast, our approach, flashpca, is based on careful combination of randomized algorithms for PCA together with parallelization, and allows the analyst to easily perform PCA on large datasets consisting of many thousands of individuals in a matter of minutes to hours. Despite relying on an approximation strategy, this approach suffers from essentially no loss in accuracy for the top eigenvalues/eigenvectors compared with traditional approaches. One practical limitation of the current implementation of flashpca is its memory requirements for large datasets: using 15,000 individuals with 43K SNPs requires ∼14 GiB RAM, and 150,000 requires ∼145 GiB. Future work will involve reducing these memory requirements without incurring a substantial performance penalty. Randomized PCA also provides the potential to de-correlate samples (“whitening”), thus essentially removing the effects of population from data prior to further downstream association analysis [9]; this will be examined in future work. It has been shown that standard PCA may be an inconsistent estimator of the true principal components in certain high dimensional settings [10], and there may be benefit from utilizing other approaches such as sparse (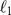-penalized) PCA [11], [12]; however, standard PCA remains widely used in practice and flashpca provides an effective way to perform such routine analyses highly efficiently.

More generally, the approach behind flashpca could prove useful for accelerating other methods that depend on performing a large number of eigen-decompositions across many samples, such as varLD [13] which assesses local differences in SNP LD between populations or FaST-LMM which implements linear mixed models of SNP data [14].

## Materials and Methods

### Ethics

All subjects included in the celiac disease dataset provided written and informed consent. For details, see the original publication [8].

### Datasets

#### HapMap3

The HapMap phase 3 dataset consists of 1184 human individuals across 11 populations (ASW: African ancestry in Southwest USA; CEU: Utah residents with Northern and Western European ancestry from the CEPH collection; CHB: Han Chinese in Beijing, China; CHD: Chinese in Metropolitan Denver, Colorado; GIH: Gujarati Indians in Houston, Texas; JPT: Japanese in Tokyo, Japan; LWK: Luhya in Webuye, Kenya; MEX: Mexican ancestry in Los Angeles, California; MKK: Maasai in Kinyawa, Kenya; TSI: Toscani in Italia; YRI: Yoruba in Ibadan, Nigeria) assayed for 1,440,616 SNPs [6]. We performed QC on the data, including removal of SNPs with MAF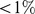, missingness 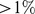, and deviation from Hardy-Weinberg equilibrium 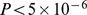. We removed non-founders and individuals with genotyping missingness 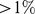, leaving 957 individuals. Next, we removed several regions of high LD and/or known inversions (chr5∶44 Mb–51.5 Mb, chr6∶25 Mb–33.5 Mb, chr8∶8 Mb–12 Mb, chr11∶45 Mb–57 Mb) [15]. Finally, we used PLINK [16] –indep-pairwise 1000 10 0.02 to thin the SNPs by LD (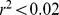), leaving 14,389 SNPs.

#### Celiac disease immunochip

The celiac disease Immunochip dataset [8] consists of 16,002 case/control individuals of British descent, assayed for 139,553 SNPs. The QC has been previously described [8]. In addition, we removed SNPs with MAF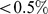 and non-autosomal SNPs, leaving 115,746 SNPs. Next, we removed the same four regions as for the HapMap3 data, and finally, we thinned the SNPs by LD with PLINK –indep-pairwise 50 10 0.5, leaving 43,049 SNPs.

### Principal Component Analysis

We represent the genotypes as an 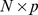 matrix 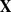, where the 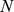 samples index the rows and the 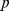 SNPs index the columns. We denote the transpose of the matrix as 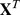. For the following, we assume that the matrix 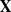 has been centered so that the mean of each column 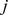 is zero (see below for variants on this).

PCA relies on finding the eigenvectors of the 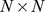 covariance matrix 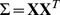 (for notational clarity we will ignore the scaling 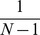 factor which can be accounted for later). This decomposition is performed using either the singular value decomposition (SVD) of the matrix 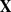 or the eigen-decomposition of the covariance matrix itself.

The SVD of 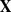 is
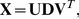
(1)where 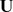 an is an 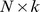 matrix (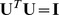) the columns of which are the eigenvectors of 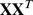, 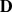 is a 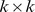 diagonal matrix of singular values (square root of the eigenvalues of 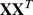 and 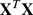), and 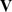 is a 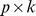 matrix (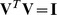) of the eigenvectors of 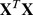, where 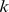 is the matrix rank (this SVD is also called the “economy SVD”). Note that SVD does not require the covariance matrix 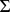 to be computed explicitly.

In the eigen-decomposition approach, the covariance matrix 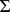 is first explicitly computed, then the eigen-decomposition is performed such that
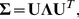
(2)where 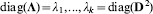 are the eigenvalues and 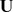 is the matrix of eigenvectors as before.

The principal components (PCs) 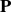 of the data are given by the projection of the data onto the eigenvectors
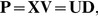
(3)where we usually truncate the matrix 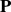 to have as many columns as required for any down-stream analysis (say, 10).

Note that some tools, such as smartpca and shellfish, output the eigenvectors 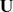 as the principal components without weighting by the singular values 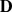, leading to different scales for the PCs. In addition, since the (empirical) covariance is typically scaled by a factor of 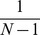, then in order to maintain the interpretation of the singular values 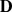 as the square-root of the eigen-values of the scaled covariance 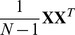, the singular values must be scaled by a factor of 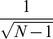 as well (as implemented in R’s prcomp). Note, however, that these scale differences have no effect on the interpretation of the principal components for ascertaining or correcting for potential population structure in data.

In traditional PCA, such as that implemented in R’s prcomp, prior to running the SVD/eigen-decomposition itself, the matrix 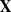 is first mean-centered by subtracting the per-column (SNP) average from each column. In contrast, smartpca [1], first centers the data, then divides by a quantity proportional to the standard deviation of a binomial random variable with success probability 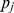


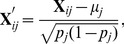
(4)where 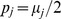 and 
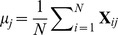
. To maintain compatibility with smartpca, flashpca employs the same standardization method by default (other scaling methods are available as well).

### Fast Principal Component Analysis

Performing PCA on large matrices (with both 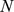 and 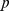 large), is time consuming with traditional approaches. To enable fast PCA, we employ an algorithm based on a randomized PCA approach [5]. Briefly, randomized PCA relies on first constructing a relatively small matrix that captures the top eigenvalues and eigenvectors of the original data, with high probability. Next, standard SVD or eigen-decomposition is performed on this reduced matrix, producing near identical results to what would have been achieved using a full analysis of the original data. Since in most genomic applications we are interested only in a few of the top eigenvectors of the data (typically 10), this allows reduction of the data to a substantially smaller matrix, and the computational cost of decomposing this matrix is negligible (see Table 1: Algorithm 1). (Note, however, that this method is general and is just as useful for extracting a much larger number of PCs).

**Table 1:** Algorithm 1.

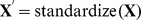
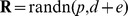
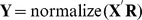
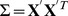
**for** *iter = 1:maxiter* **do**
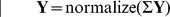
**end**
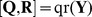
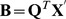
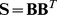
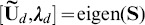
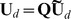
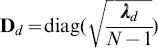
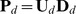

Pseudocode for the eigen-decomposition variant of the fast PCA, based on the randomized algorithm of [5] for the case where 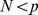. 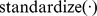 is the standardization in Equation 4. 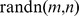 is a function generating an 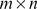 iid multivariate normal matrix, 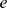 is the user-defined number of extra dimensions, 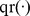 is the QR decomposition, 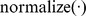 is a function that divides each column 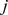 by its 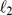 norm 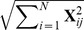, 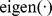 is the eigen-decomposition producing the 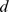-top eigenvectors 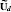 and vector of 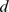-top eigenvalues 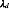. 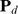 is the matrix of 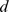 principal components.

Focusing on the eigen-decomposition approach (Equation 2), the two main computational bottlenecks are (i) computing the 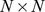 covariance matrix 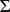 and (ii) when 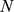 is large, the eigen-decomposition step itself. In our fast PCA approach, the first bottleneck cannot be avoided but can be mitigated through parallel computation (see Implementation). The second bottleneck is circumvented via the randomized approach, by constructing a matrix 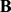 of size 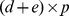, from which the small 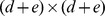 matrix 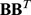 is formed and used for the eigen-decomposition, where 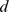 is the number of required eigenvectors (say 10), and 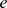 is the number of auxiliary dimensions used to increase accuracy which can be discarded later. We have found that a total dimension of 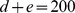 is more than sufficient for producing good results in the first 10 PCs; hence the eigen-decomposition need only be performed on 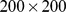 matrix while producing near identical results to a full PCA on the original data.

Another computational shortcut for the case where 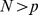, applying equally to PCA and to randomized PCA, is simply transposing the data 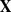, then standardizing the rows instead of the columns. An identical PCA algorithm is then run on the transposed data, with the only difference being that the estimated matrix 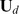 will now contain the top-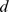 eigenvectors of 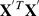 instead of 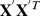, and hence the final 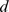 principal components will be 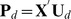. This procedure makes it possible to analyze large datasets with 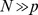 at a cost not much greater than when 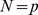.

While the SVD approach is generally recommended for reasons of better numerical stability and speed, we have found that when 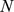 and 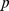 are in the thousands, the above eigen-decomposition approach is substantially faster since the matrix multiplication is trivially parallelizable (and is parallelized in practice in flashpca), allowing the decomposition to be performed on the small matrix 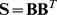 which is of size 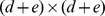 rather than a more expensive non-parallelized SVD of the matrix 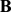, which is an 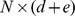 matrix, with no discernible effect on accuracy of the top principal components; however, both methods are implemented in flashpca (yet another possibility is to perform SVD on 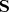, but this is not implemented yet).

### Implementation

flashpca is implemented in C++ and relies on Eigen [17], a C++ header-only library of numerical linear algebra algorithms, which allows for native parallelization of certain computations through OpenMP threads when multiple CPU cores are available. flashpca natively reads PLINK [16] SNP-major BED files, avoiding the need to convert these files to other formats. flashpca is licensed under the GNU Public License (GPL) v3; source code and documentation are available at https://github.com/gabraham/flashpca.

Prior to PCA, thinning of the SNPs by LD and removal of regions with known high LD or other artefacts such as inversions have been recommended [1], [15], as high correlations between the SNPs can distort the resulting eigenvectors. For this purpose we recommend using PLINK v2 (https://www.cog-genomics.org/plink2) which is substantially faster than PLINK v1.07. Reducing a dataset to ∼10,000–50,000 SNPs is usually sufficient to achieve an accurate PCA, and can be done using –indep-pairwise.

## Supporting Information

File S1 Concordance in principal components 1–10 between smartpca, flashpca, shellfish, and R’s prcomp on the HapMap3 dataset.
(PDF)

## References

[1] Price AL, Patterson NJ, Plenge RM, Weinblatt ME, et al (2006) Principal components analysis corrects for stratification in genome-wide association studies. Nat Genet 38: 904–909. 16862161 10.1038/ng1847

[2] Patterson N, Price AL, Reich D (2006) Population Structure and Eigenanalysis. PLoS Genet 2: e190. 17194218 10.1371/journal.pgen.0020190 PMC1713260

[3] Novembre J, Johnson T, Bryc K, Kutalik Z, Boyko AR, et al (2008) Genes mirror geography within Europe. Nature 456: 98–101. 18758442 10.1038/nature07331 PMC2735096

[4] Halko N, Martinsson PG, Shkolnisky Y, Tygert M (2011) An Algorithm for the Principal Component Analysis of Large Data Sets. SIAM Journal on Scientific Computing 33: 2580–2594.

[5] Halko N, Martinsson PG, Tropp JA (2011) Finding Structure with Randomness: Probabilistic Algorithms for Matrix Decompositions. SIAM Review 53: 217–288.

[6] International HapMap 3 Consortium (2010) Integrating common and rare genetic variation in diverse human populations. Nature 467: 52–58. 20811451 10.1038/nature09298 PMC3173859

[7] R Development Core Team (2011) R: A Language and Environment for Statistical Computing. R Foundation for Statistical Computing, Vienna, Austria. Avaliable: http://www.R-project.org. ISBN 3-900051-07-0.

[8] Trynka G, Hunt KA, Bockett NA, Romanos J, Mistry V, et al (2011) Dense genotyping identifies and localizes multiple common and rare variant association signals in celiac disease. Nat Genet 43: 1193–201. 22057235 10.1038/ng.998 PMC3242065

[9] Rakitsch B, Lippert C, Stegle O, Borgwardt K (2013) A Lasso Multi-Marker Mixed Model for Association Mapping with Population Structure Correction. Bioinformatics 2: 206–214. 10.1093/bioinformatics/bts669 23175758

[10] Johnstone IM, Lu AY (2009) On Consistency and Sparsity for Principal Components Analysis in High Dimensions. Journal of the American Statistical Association 104: 682–693. 20617121 10.1198/jasa.2009.0121 PMC2898454

[11] Zou H, Hastie T, Tibshirani R (2006) Sparse Principal Component Analysis. Journal of Computational and Graphical Statistics 15: 265–286.

[12] Lee S, Epstein MP, Duncan R, Lin X (2012) Sparse Principal Component Analysis for Identifying Ancestry-Informative Markers in Genome-Wide Association Studies. Genetic Epidemiology 302: 293–302. 10.1002/gepi.21621 PMC3596262 22508067

[13] Ong RTH, Teo YY (2010) varLD: a program for quantifying variation in linkage disequilibrium patterns between populations. Bioinformatics 26: 1269–70. 20308177 10.1093/bioinformatics/btq125

[14] Lippert C, Listgarten J, Liu Y, Kadie CM, Davidson RI, et al (2011) FaST linear mixed models for genome-wide association studies. Nature Methods 8: 833–5. 21892150 10.1038/nmeth.1681

[15] Fellay J, Ge D, Shianna K, Colombo S, Ledergerber B, et al (2009) Common Genetic Variationand the Control of HIV-1 in Humans. PLoS Genet 5: e1000791. 20041166 10.1371/journal.pgen.1000791 PMC2791220

[16] Purcell S, Neale B, Todd-Brown K, Thomas L, Ferreira MA, et al (2007) PLINK: a tool set for whole-genome association and population-based linkage analyses. Am J Hum Genet 81: 559–575. 17701901 10.1086/519795 PMC1950838

[17] Guennebaud G, Jacob B (2010). Eigen v3. Avaliable: http://eigen.tuxfamily.org.

