# Supplementary figures and images for: Fast Principal Component Analysis of Large-Scale Genome-Wide Data
Source: PLoS One. 2014 Apr 9;9(4):e93766. doi: 10.1371/journal.pone.0093766 (PMC3981753; doi:10.1371/journal.pone.0093766)

# PC 1

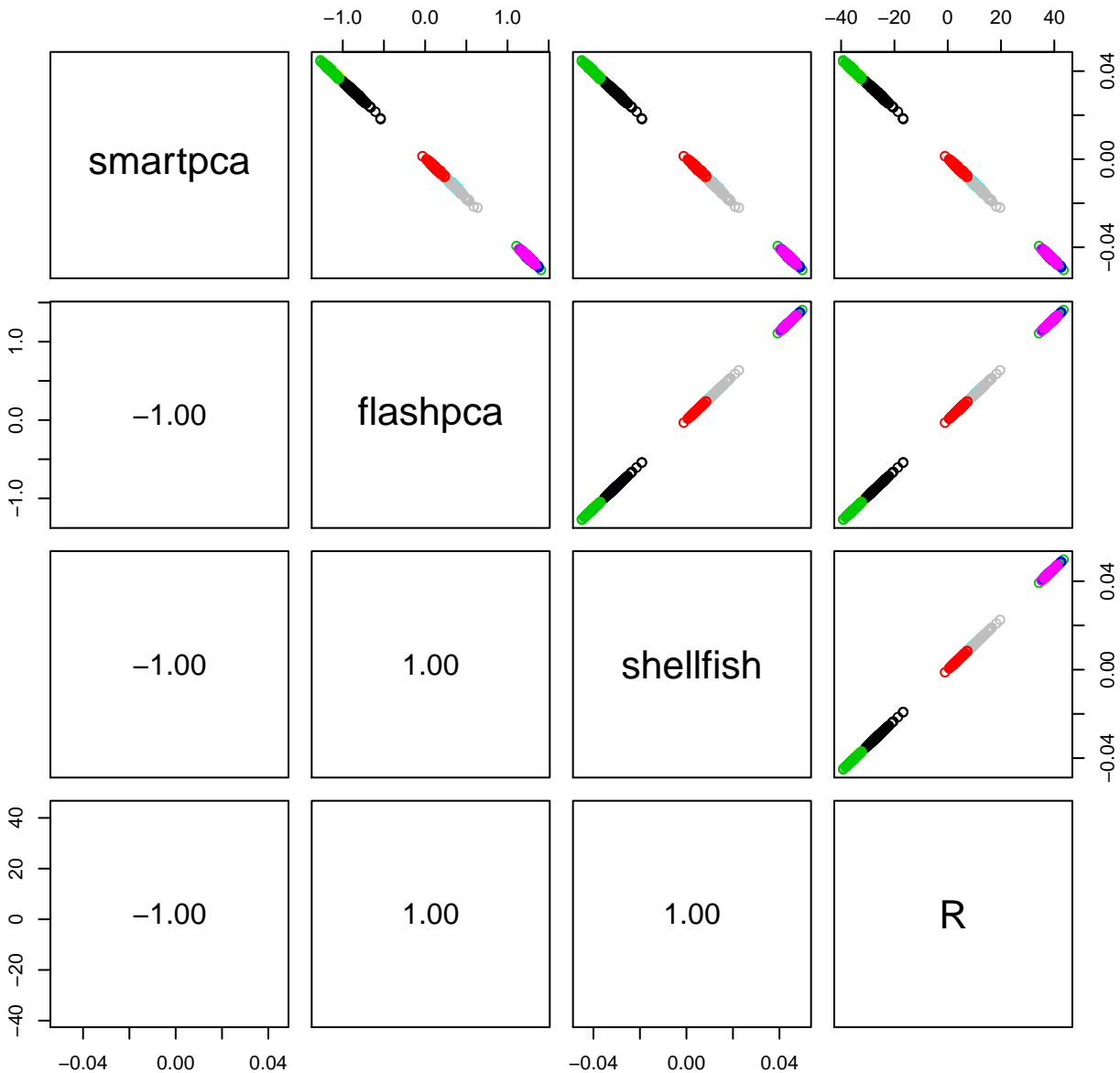

## PC 2

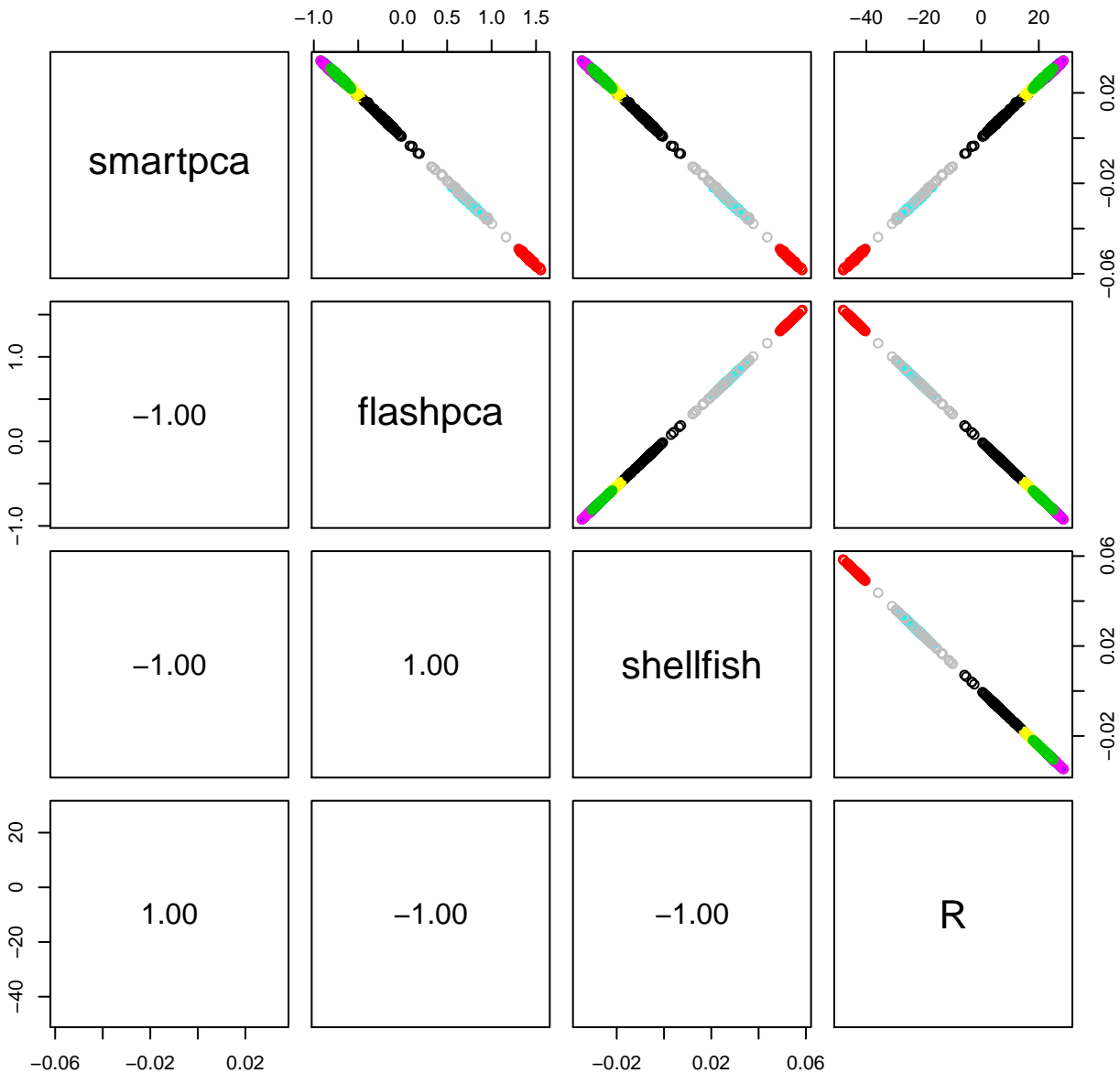

# PC 3

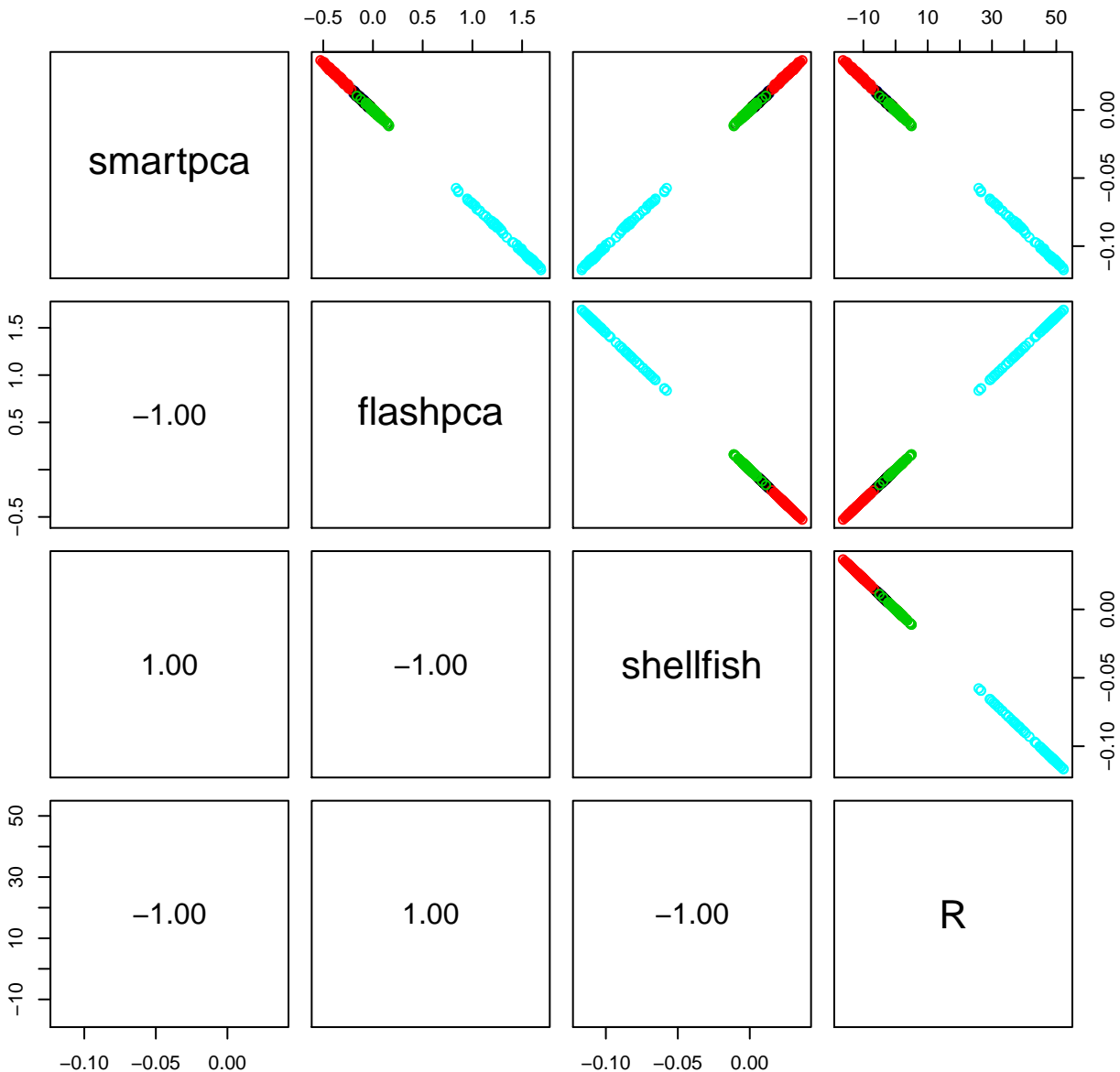

# PC 4

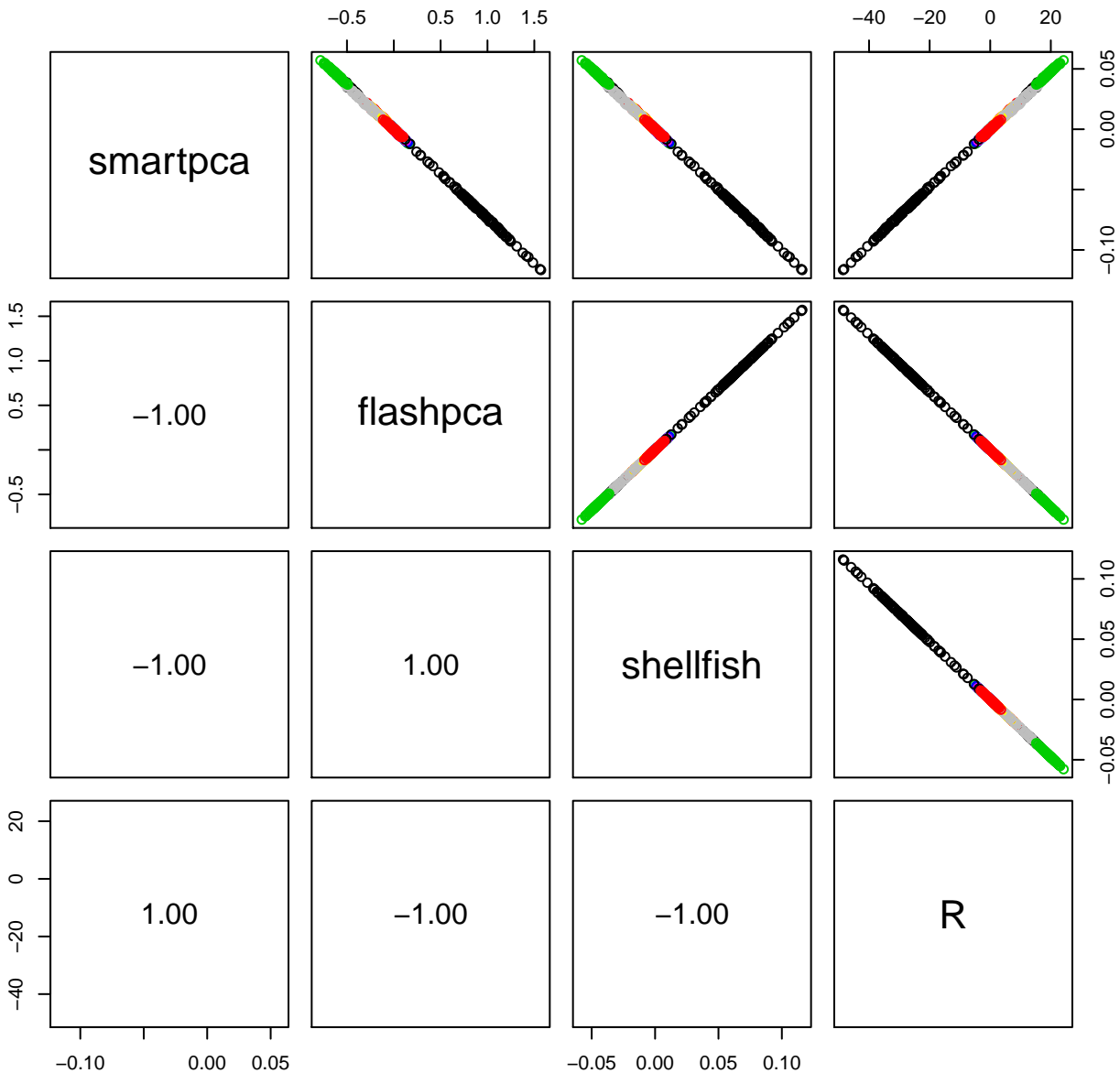

# PC 5

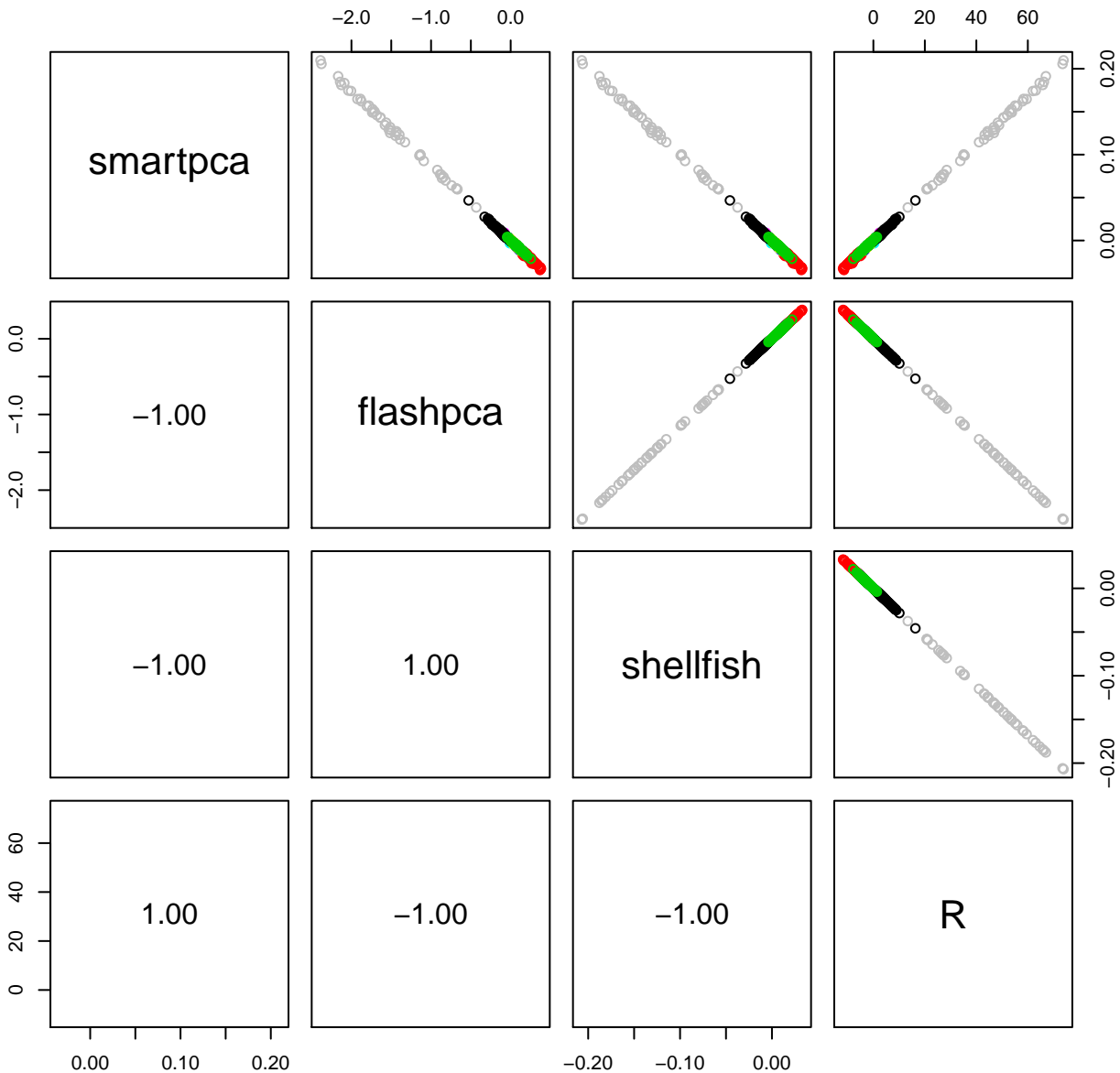

# PC 6

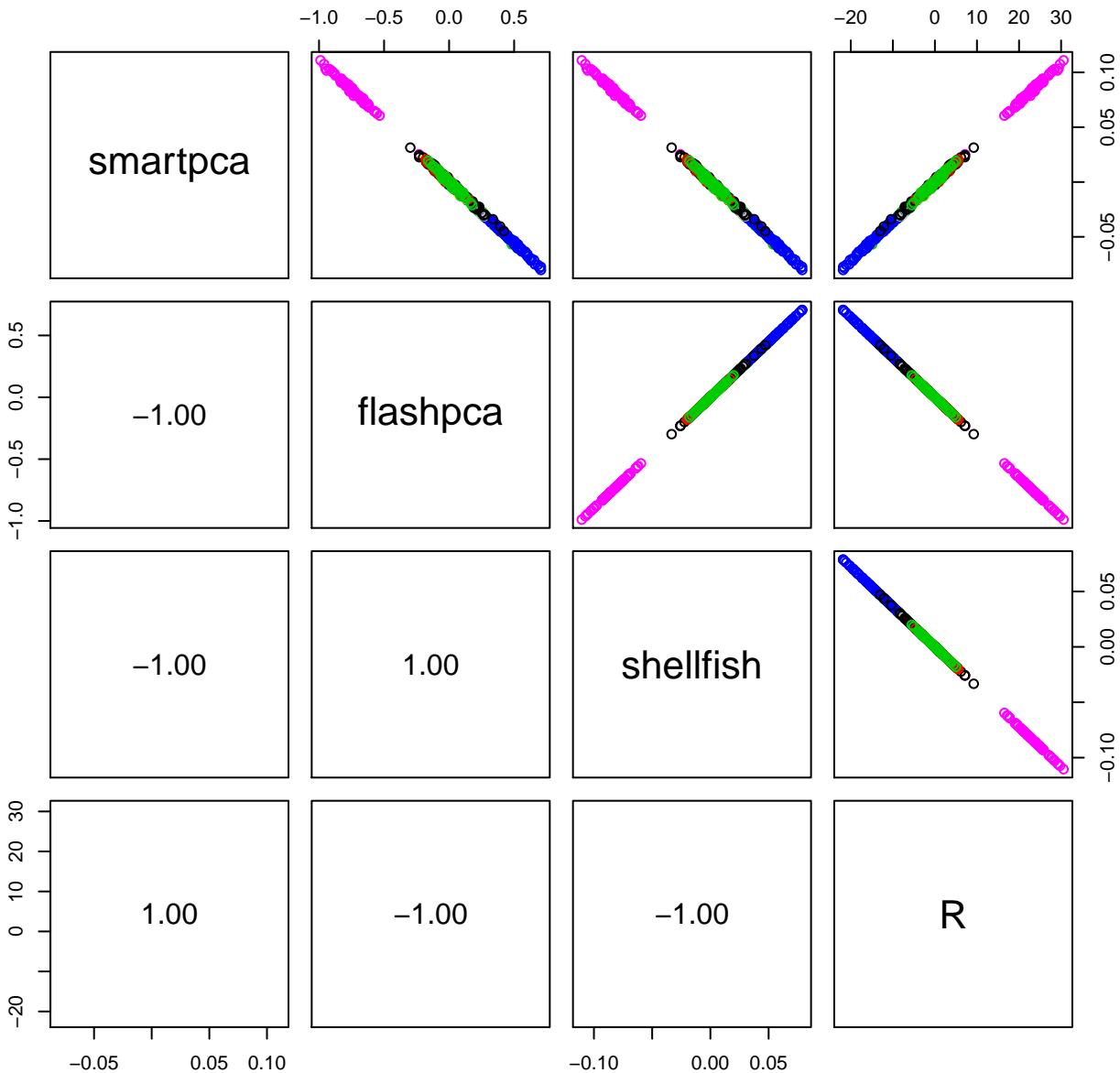

# PC 7

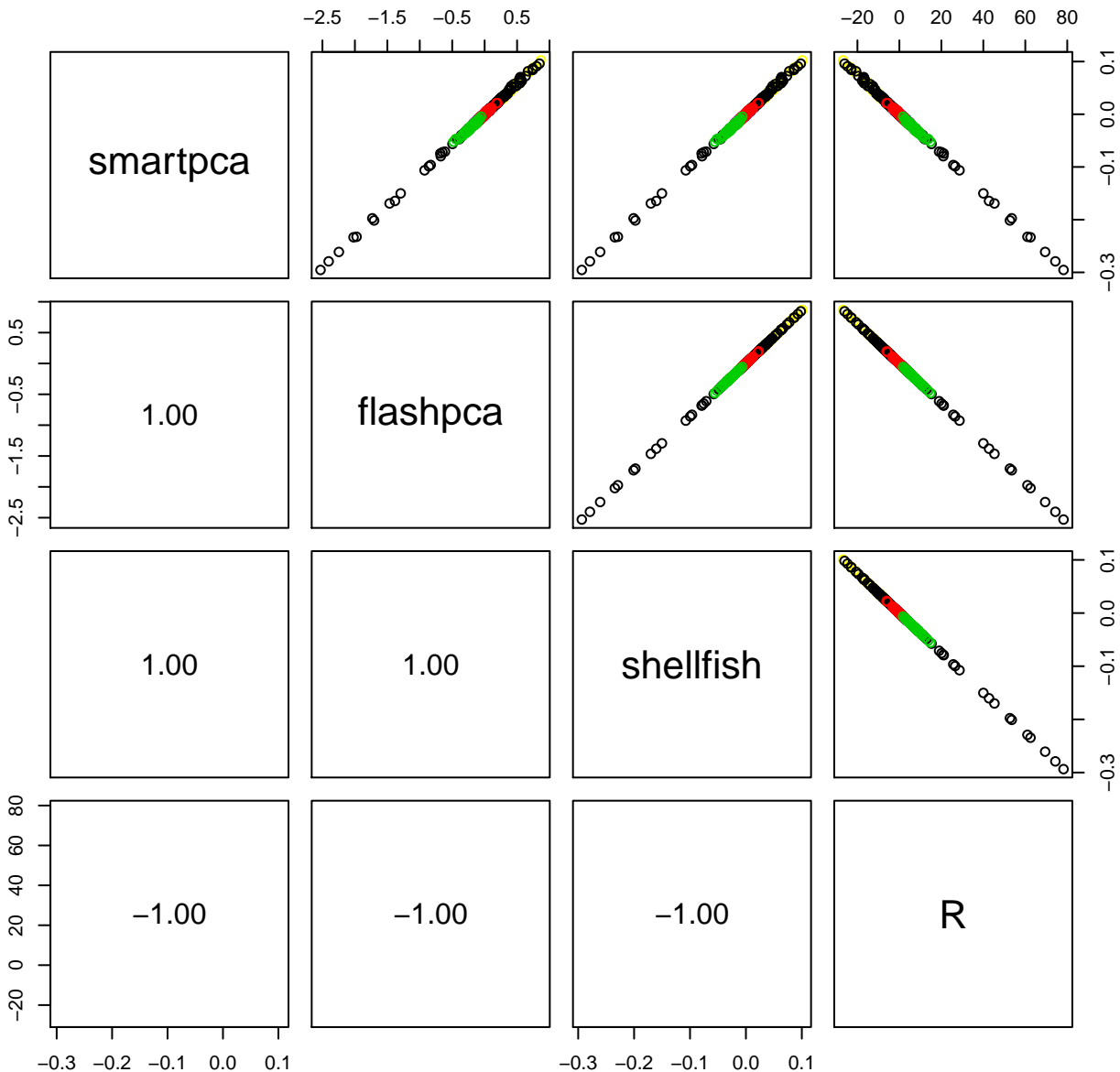

# PC 8

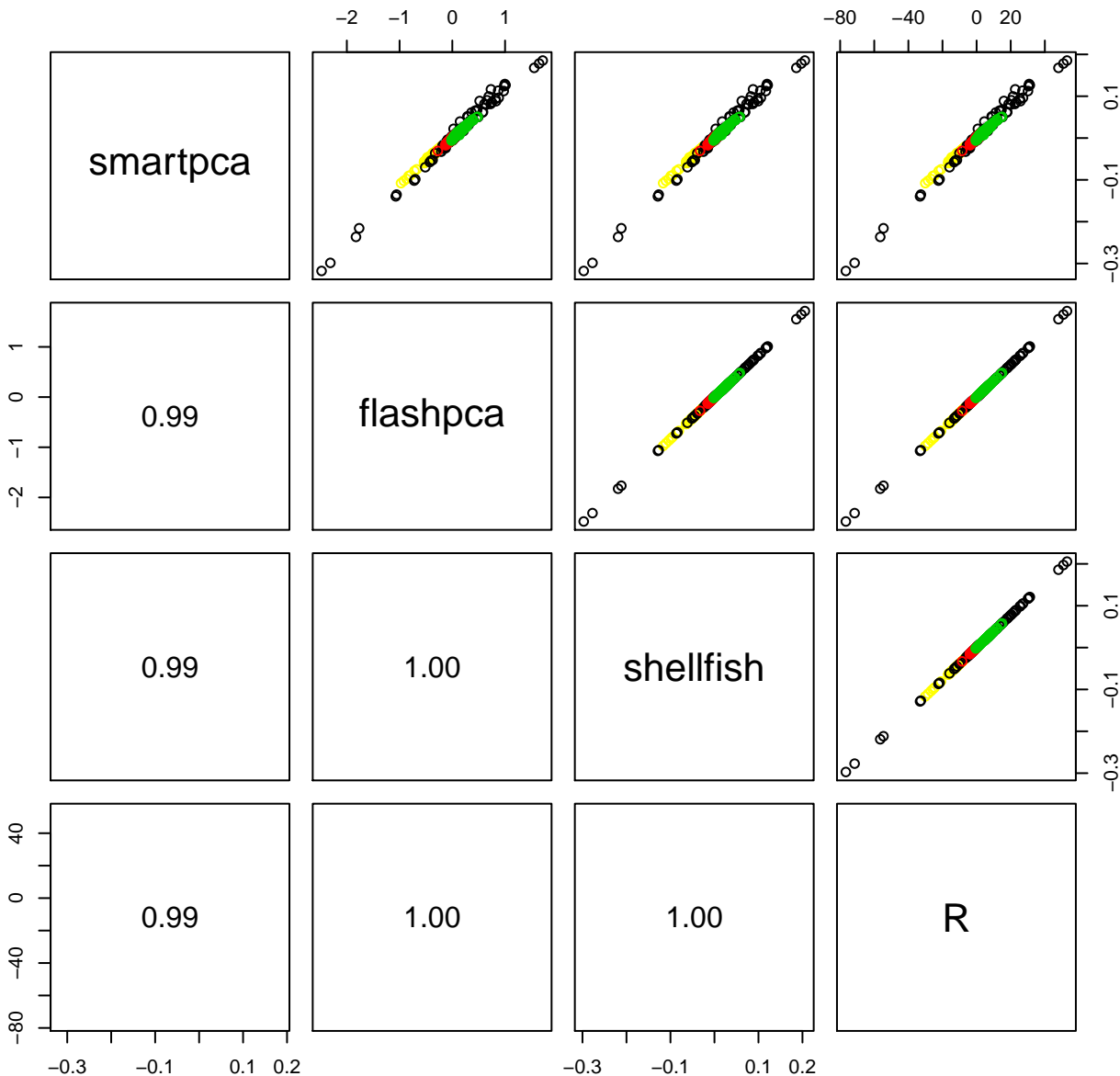

# PC 9

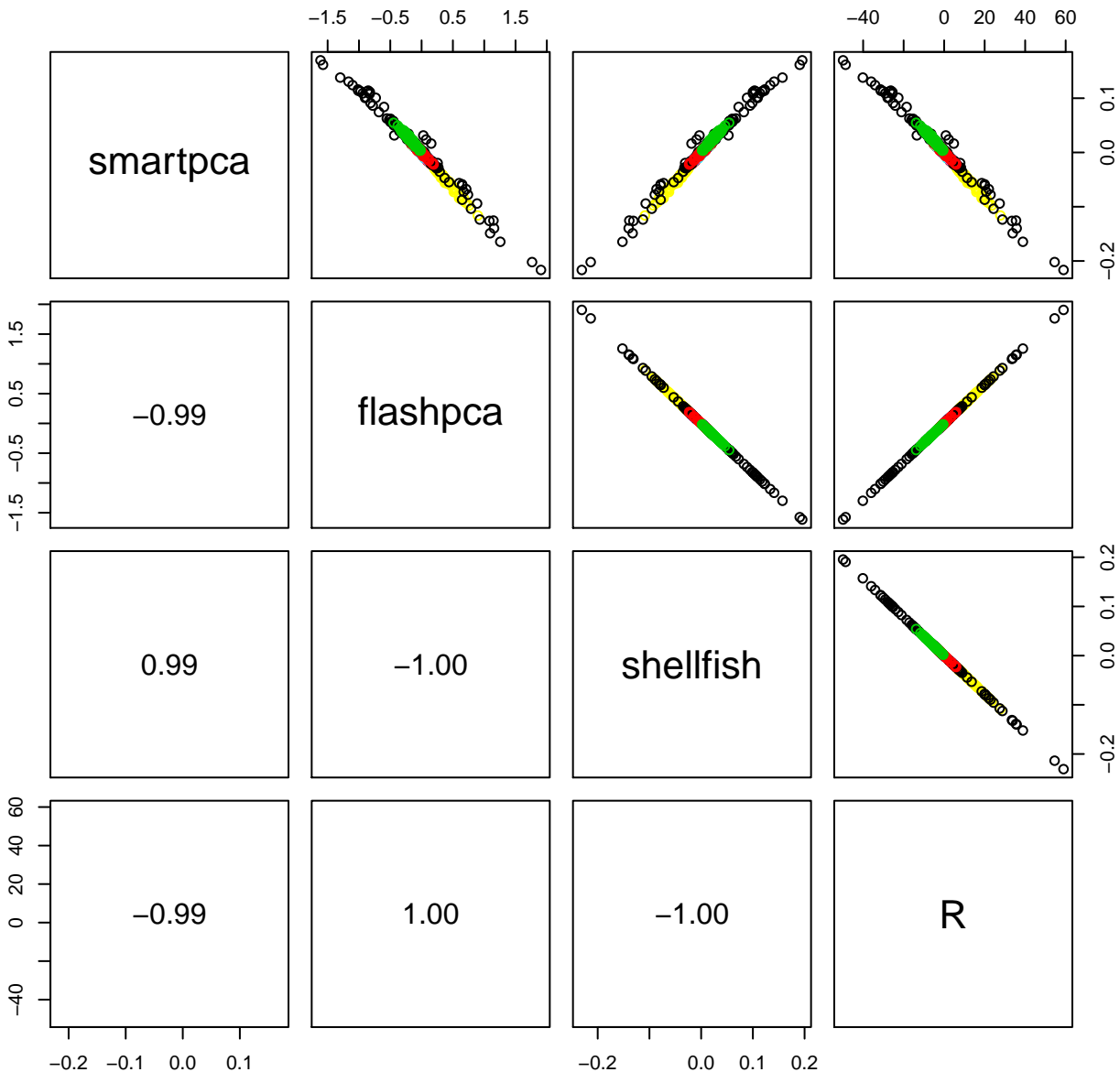

# PC 10

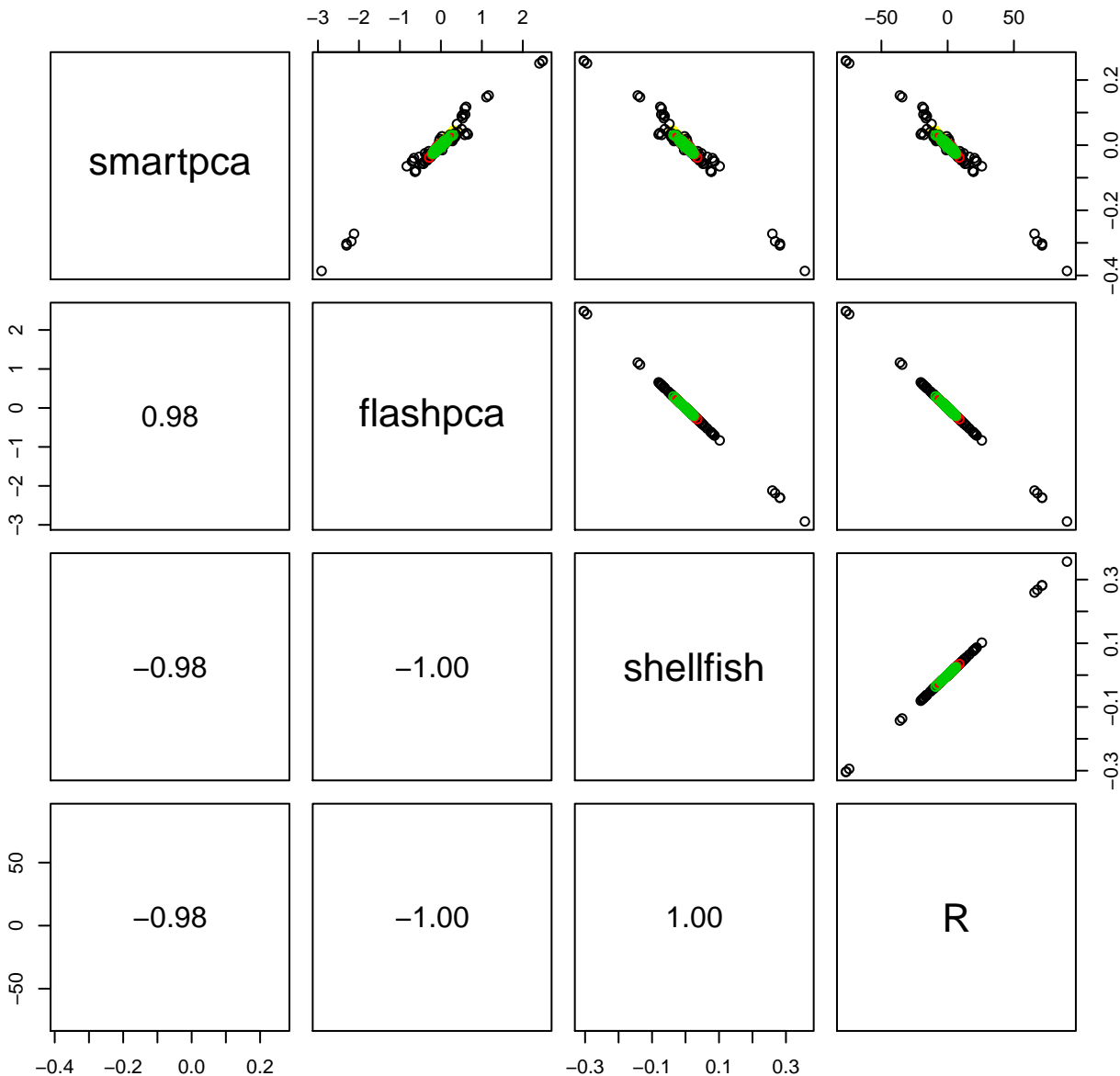

Supplement: File S1 — Concordance in principal components 1–10 between smartpca, flashpca, shellfish, and R’s prcomp on the HapMap3 dataset. (PDF) [file pone.0093766.s001.pdf]
